# Full data acquisition in Kelvin Probe Force Microscopy: Mapping dynamic electric phenomena in real space

**DOI:** 10.1038/srep30557

**Published:** 2016-08-12

**Authors:** Liam Collins, Alex Belianinov, Suhas Somnath, Nina Balke, Sergei V. Kalinin, Stephen Jesse

**Affiliations:** 1 Center for Nanophase Materials Sciences, Oak Ridge National Laboratory, Oak Ridge, Tennessee 37831, USA; 2 Institute for Functional Imaging of Materials, Oak Ridge National Laboratory, Oak Ridge, Tennessee 37831, USA

## Abstract

Kelvin probe force microscopy (KPFM) has provided deep insights into the local electronic, ionic and electrochemical functionalities in a broad range of materials and devices. In classical KPFM, which utilizes heterodyne detection and closed loop bias feedback, the cantilever response is down-sampled to a single measurement of the contact potential difference (CPD) per pixel. This level of detail, however, is insufficient for materials and devices involving bias and time dependent electrochemical events; or at solid-liquid interfaces, where non-linear or lossy dielectrics are present. Here, we demonstrate direct recovery of the bias dependence of the electrostatic force at high temporal resolution using General acquisition Mode (G-Mode) KPFM. G-Mode KPFM utilizes high speed detection, compression, and storage of the raw cantilever deflection signal in its entirety at high sampling rates. We show how G-Mode KPFM can be used to capture nanoscale CPD and capacitance information with a temporal resolution much faster than the cantilever bandwidth, determined by the modulation frequency of the AC voltage. In this way, G-Mode KPFM offers a new paradigm to study dynamic electric phenomena in electroactive interfaces as well as a promising route to extend KPFM to the solid-liquid interface.

Progress in high-resolution imaging techniques such as Kelvin probe force microscopy (KPFM)^1^, a variant of atomic force microscopy (AFM)^2^, has allowed imaging of ***static*** electronic and electrochemical surface properties^3^,^4^,^5^. Originally developed to measure the contact potential difference (CPD) between a conducting AFM tip and a metallic sample^1^, the technique has been extended further to semiconductors^5^,^6^, operational devices^7^,^8^,^9^, insulators^10^, ferroelectrics^11^,^12^,^13^, and ion conductors^14^. Despite this broad applicability, the fundamental operational principles and the character of the derived information has not changed from the original concepts outlined by Nonnenmacher *et al*.^1^.

Quantitative interpretation of KPFM images hinges on several key assumptions such as: (i) a linear, lossless, dielectric between tip and sample, (ii) stable electronic properties on the timescale of the measurement, (∼1–3 ms) as well as (iii) absolute compensation of the electrostatic force and optimal feedback operation. Noteworthy, for electroactive materials involving ionic or charge transport^15^,^16^, or operation in electrolytes containing mobile ions^17^, the first two requirements for quantitative KPFM can readily breakdown. Equally, for a poorly tuned measurement (e.g. incorrect phase settings and feedback gains^18^, parasitic influences^19^,^20^,^21^ etc.) the measured CPD can deviate by ∼100’s of millivolts from the true CPD in an instrument specific fashion^22^,^23^,^24^. In addition, the closed loop nature of the classical KPFM approach makes it impossible to verify the veracity of the underlying assumptions^23^. As such, Kelvin probe force spectroscopy (KPFS) is often preferred for precise measurements of CPD^3^. KPFS consists of applying a slow varying DC bias, to the tip, located above a single spatial location, while monitoring the dynamic response of the cantilever using heterodyne detection. This measurement paradigm involves long integration times (100 s of μs to ms) and when operated in a spectroscopic mode consumes a significant amount of time to collect high resolution images. As an example, the now famous paper by Mohn *et al*.^3^ which used KPFS to map the charge distribution in a single molecule took 33 hours to collect and hence required drift correction. Notably, KPFS does overcome the requirement for bias feedback, and allows the veracity of the electrostatic interaction to be assessed from the quality of the parabolic fit. Indeed, it has been demonstrated that the dynamic transients, or abrupt deviations from the simple capacitor model which can be caused by tunneling events, charging, and charge relaxation; all of which can be identified as deviations from a purely parabolic voltage dependence^25^,^26^,^27^.

The interest in fast local charging, or ion dynamics has been recognized within the KPFM community and resulted in the development of novel time resolved KPFM modes (or related variants such as Electrostatic Force Microscopy (EFM)) that can capture temporally resolved maps of electrochemical dynamics from millisecond^16^,^28^ to nanosecond timescales^29^,^30^. These approaches provide information beyond the time averaged CPD, and have proven useful in probing the time dependent ionic transport in lateral devices^14^, surface photovoltage and charge carrier generation in photovoltaic materials^15^,^31^, as well as charge screening and ion dynamics at the solid-liquid interface^17^,^32^. Time resolved EFM (tr-EFM)^15^ was developed to measure photoexcited charge in polymer films with a lateral resolution of 100 nm and temporal resolution on the order of 100 μs. This approach has proven particularly useful for making local quantum efficiency maps as a function of material properties and preparation^15^, photodegradation^31^,^33^, and excitation wavelength^33^. Unfortunately, the measurement time resolution is limited by the bandwidth of the phase locked loop in the tr-EFM setup^29^. More recently, the same group has circumvented this bottleneck by developing a fast tr-EFM which avoids heterodyne detection and allows subcycle detection of dynamic events through analysis of the raw photodetector stream^29^. This approach however cannot be used to extract quantitative information on electrochemical potentials directly, as it focuses on the detection of rise times, which are then related to fast charge dynamics via a calibration curve^29^. Pump-probe KPFM^34^ has been demonstrated to simultaneously detect the time averaged CPD and nanosecond changes in surface charges. This approach has been used to spatially map “speed bumps” in organic field-effect transition devices^35^. Importantly, this technique still relies on single frequency heterodyne detection and bias feedback, and hence is subject to the standard assumptions required for KPFM operation, albeit with the added benefit of temporal dynamic information. Finally, these approaches rely on an *assumed* functional form of the excitation time event, in order to understand the dynamic response.

Recently we have developed a technique called General Acquisition Mode (G-Mode)^36^,^37^,^38^ SPM which allows full exploration of the cantilever deflection with extremely high temporal resolution. G-Mode works by capturing, storing and compressing the AFM photodetector signal at the sampling rate limit (∼4 MHz) providing a densely sampled permanent record of the dynamic cantilever trajectory. The G-Mode approach has been applied to tapping mode AFM^36^, piezoresponse force microscopy (PFM)^37^, Magnetic force microscopy (MFM)^39^ and dual harmonic-KPFM^38^. To date, G-Mode KPFM has been demonstrated as an alternative to traditional heterodyne detection and bias feedback approaches, at the cost of large multidimensional data sets. While in the early 90’s, as KPFM emerged, storing multi gigabyte (pre-compression) files per experiment was inconceivable, today these file sizes are becoming routine^40^. G-Mode KPFM has been shown to be capable of emulating classical KPFM, while also allowing additional flexibility in terms of noise exploration in the frequency, and the spatial domains, as well as multiple information channel capture^38^,^39^.

In this work, we demonstrate analysis of the time-dependent G-Mode KPFM response involving both physical and information based analysis approaches. We outline a procedure to recover the clean electrostatic response from the noisy photodetector signal. The raw photodetector signal is processed in such a way as to preserve the temporal information encoded in the cantilever response, typically sacrificed by signal averaging. Using physics based analysis; we show that G-Mode KPFM allows the parabolic bias dependence of the electrostatic force to be recovered for each period cycle of the AC voltage, leading to spatial and temporal dependence of the CPD and capacitance information channels. We illustrate that this methodology can allow fast KPFM measurements with a temporal resolution substantially faster than the cantilever bandwidth, determined by the modulation frequency of the AC voltage alone (e.g. 66 μs in this work). Furthermore, we demonstrate that multivariate statistical methods can easily separate the complicated multidimensional data sets into statistically significant components, and extract or help visualize hidden information on time dependent phenomena, which can be mapped onto individual physical mechanisms in a fast and computationally efficient manner^40^,^41^,^42^.

We begin by exploring the information transfer in traditional KPFM. The relevant aspects of KPFM operation are demonstrated schematically in Fig. 1a. Common to all modes of KPFM is the applied tip voltage, shown in Equation 1:


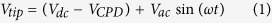


here, *V*_*ac*_ is a sinusoidal voltage modulation at the excitation frequency (*ω*), *V*_*dc*_ is a backing voltage typically controlled by the bias feedback loop, and *V*_*cpd*_ is the CPD between the probe and the tip. Assuming a linear lossless dielectric in the tip-sample junction, the electrostatic force is described by Equation 2


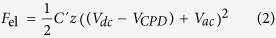


where, *C′*_*z*_ is the capacitance gradient, which is dependent on the geometry and the dielectric properties of the tip-sample capacitor. The total electrostatic force comprises of a static DC capacitive force, (*F*_*dc*_) as well as forces at the excitation frequency (F_*ω*_) and its harmonic (F_2*ω*_). Detection of these weak long range electrostatic forces, however, presented a significant technical hurdle in the early implementations of KPFM^1^, ultimately leading to adoption of heterodyne detection. This introduced an important tradeoff between noise attenuation and meaurement response time dictated by the low-pass filter bandwidth and rolloff of the lock in amplifier (or phase locked loop). Namely, a narrower bandwidth will remove noise very close to the reference frequency but will increase the measurement time constant. Classical KPFM utilizes this approach to detect and sequentially compensate for the first harmonic response of the cantilever, while attenuating all other responses, leading to a complete loss of all information outside the modulation frequency (e.g. information on polarization forces and dielectric properties encoded in the cantilever response (e.g. F2w))^43^,^44^,^45^. In KPFM, the detected first harmonic amplitude is supplied to a bias feedback loop which continually adjust *V*_*dc*_ to nullify the electrostatic force, further increasing the measurement time constant. The *V*_*dc*_ required to eliminate the electrostatic force is assumed to be equal to the *V*_*cpd*_ (note the characteristic 1/*V*_*ac*_ errors in this scheme^46^), which results in a 2D map of spatial variation in the time averaged CPD.

From the vantage of information transfer, KPFM reduces the ∼1–10 MHz data stream from the photodetector to a matrix of CPD values in which the compression is chosen to match the acquisition time of single spatial location. Importantly, the closed-loop nature of KPFM also means that important information related to the bias dependence of the electrostatic force is lost, and the assumption that the system behaves like an ideal capacitor with a parabolic bias dependence of the electrostatic force is postulated rather than proven. Clearly, in the presence of fast dynamics (i.e. faster or on the scale of the measurement time) the response may deviate from a parabolic bias dependence^25^,^26^,^27^.

G-Mode KPFM, on the other hand, does not use heterodyne detection or bias feedback and allows analysis of the raw cantilever deflection stream directly. All information regarding the spatial and time/frequency response of the vertical displacement at the end of the cantilever as a function of the applied AC voltage, can be recovered. This data can be accessed and analysed without any effective information pre-processing (imposed by the averaging of the heterodyne methods) maintaining the effective veracity of the original photodetector signal. For data capture, an arbitrary waveform generator and digitizer (National Instruments PXIe 6124) is used to modulate the tip electrically as well as to capture the photodetector response. In the G-Mode measurements shown here, a sinusoidal voltage waveform with a frequency (15 kHz) well below half the first resonance peak (∼70 kHz) was applied to the cantilever. Measurements were performed during the raised portion of a lift (or dual pass) mode, where the topography is measured during the first trace and retrace, and KPFM is done across the same line, but with a 50 nm offset from the surface. This mode limits the tip-surface interactions to long range electrostatic forces. The processing and analysis procedure in G-Mode KPFM is shown in Fig. 1b. A typical raw data file size for a 256 by 256 pixels image captured at a scan rate of 0.45 Hz (approx. 18 mins per scan) is 4 GB. After storage, digital signal processing techniques are used to isolate the electrostatic response from the background via noise filtering. We transform the cantilever response from the time into the frequency domain using a fast Fourier transform (FFT) operation. Since the entire response is preserved, the time domain response from a single pixel of data (∼4 ms), a line, (∼1.02 seconds) or the entire image itself (∼18 minutes) can be used to construct the frequency spectrum. Practically, we found that it is sufficient to read in one line of data at a time and determine the local noise floor, shown in Fig. 2(a). This frequency spectra inspection is extremely informative for processing downstream. During this first pre-processing stage, noise thresholds and optional low pass and/or band pass filtering can be applied judiciously, based on the data from the entire experiment (as opposed to on the timescale of a single pixel as is conventionally performed). After de-noising, the signal if converted back into the time domain using an inverse FFT and reshaped into a three, or higher dimensional, dataset and segmented. A slice of the filtered and unfiltered time domain response is shown in Fig. 2(b), where a combination of low-pass filters (40 kHz applied), band pass filters (0.1–5 kHz) and noise-thresholds (10 a.u.) were applied in the frequency domain (i.e. the amplitude of bins outside the filter bands, or below the noise floor are set to zero) before converting back into the time domain.

Once the data has been denoised and transformed back into the time domain, direct reconstruction of the electrostatic force versus voltage can be realised by plotting the cantilever response versus the applied voltage over one (or many) periods of oscillation, as shown in Fig. 2(c–f). To demonstrate the effectiveness of the filtering criteria on the recovered parabola, we repeated the signal processing step for different filter settings on the same data, without any distortion of the original information. Figure 2(c) shows the raw data (measured response with no filtering) plotted against the applied voltage where the response is entirely dominated by noise. After the application of a 120 kHz low pass filter and a 1 a.u. noise floor (dashed green line in Fig. 2(a)) a noisy parabola becomes apparent, see Fig. 2(d). Figure 2(e) depicts the same parabola with a low pass filter of 120 kHz applied and a 10 a.u. noise floor (dashed red line in Fig. 2(a)). Increasing the noise floor rejects much of the noise and a clearly identifiable parabola is recovered. The response does however show significant deviations from an ideal parabola as a result of the influence from the first resonance peak, which is above the noise floor threshold. Finally, Fig. 2(f) shows the resulting parabola after optimal filter settings are chosen (low pass filter: 40 kHz, band pass filter: 0.1–3 kHz, and a noise floor of 10 a.u.) such that almost all noise is rejected and the influence of the resonance peak is removed via an appropriate low pass filter. In Fig. 2 we have manually set the noise floor to demonstrate its influence on the recovered parabola, however, in practice this process is automated as outlined in the Supplementary information.

After the optimal signal processing is implemented, the full response versus voltage can be recovered for each half cycle of the applied voltage, as has been shown in Fig. 2. Noteworthy, as in all KPFM approaches, appropriate consideration should be given to the phase offset between drive and response. An unaccounted for phase offset between drive and response can result in perceived hysteresis between positive and negative voltage cycles and should be removed to ensure that accurate CPD can be quantitatively determined from the parabolic response, see Supplementary information. We fit the response by a second order polynomial curve described by y = a*x*^2^ + *bx* + *c*, where CPD = −*b*/*a*, and a is directly proportional to *C′*_*z*_ with *c* as the amplitude offset of the parabola. In summary, unlike standard KPFM which gives a readout of the time averaged CPD (∼1–3 ms) the procedure outlined here allows both CPD and *C´*_*z*_ to be determined at very high temporal resolution (τ_G-mode_ = 1/2ω).

To thoroughly understand the underlying physics and influence of experimental parameters on the time dependence of the recorded response, we compare experimental data with numerical simulations of the cantilever motion. In the simulation, the dynamics of the freely oscillating cantilever can be adequately modelled as a damped harmonic oscillator. The equation of motion including an inertial term, a damping term and a restoring force is given by Equation 3:


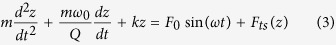


where *m* is the effective mass, ω_0_ is the mechanical resonance frequency, and *z* is the tip’s deviation from its relaxed position. The drag of the cantilever motion or the viscous damping coefficient is *mω*_*0*_/*Q*. The dimensionless quality factor, *Q*, is a measure of how fast the cantilever relaxes to equilibrium. *Q*, is defined in the frequency domain as the full width at half maximum of the resonance peak and in practical terms, it determines the cantilever bandwidth *τ*_*cant*_, as *τ*_*cant*_ = 2*Q*/ω_0_. The external forces are composed of the time-dependent excitation force *F*_0_ sin (*ωt*) and the distance-dependent tip sample force *F*_*ts*_. In our model, the behavior of the cantilever is approximated by two simple harmonic oscillators (SHO) as given by Equation 4 where only the first two eigenmodes are considered. The following expressions for the oscillation amplitude *A*_*0*_ is:


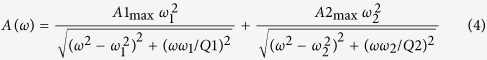


The cantilever parameters for the first and second eigenmode of the cantilever were; *ω*_1_ = 75 kHz, A1_max_ = 0.8 and *Q*1 = 100 and *ω*_2_ = 420 kHz, A2_max_ = 0.1, and *Q*2 = 180 respectively. In the simulation, we apply a single frequency sine wave voltage superimposed with a 1 V_dc_ square wave a period of 2 ms, below the cantilever bandwidth *τ*_*cant*_ = 2.8 ms. The electrostatic force was calculated using the Equation 2 where V_ac_ = 2, V_cpd_ = 0.25 V and 
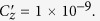
The cantilever response was found via the product of the cantilever transfer function and the electrostatic force in the frequency domain. Random white noise was added to the system and the response was inverse FFT-ed to recover the cantilever response in the time domain. The simulated excitation and time domain response of the cantilever are shown in Fig. 3(a,b) respectively. A ringdown of the cantilever response can be observed at the instant the DC pulse is applied to the tip, taking several milliseconds to equilibrate as determined by the cantilever bandwidth. In Fig. 3(c) we explore the influence of the ringdown on the recovered parabolic response by plotting the simulated response versus the applied voltage. We observed two parabolas whose maxima were separated in voltage as a result of the application of a DC bias pulse. Slight distortions in the parabola are also observed. The parabolas were fit to a second order polynomial to determine the exact CPDs, Fig. 3(d), which measured the bias pulse to be 880 mV, underestimating the 1 V pulse by 22%. This deviation is a result of the frequency dependent gain of the cantilever transfer function, known in dual harmonic-KPFM^47^, with its removal described elsewhere^23^,^38^ as well as in the Supplementary information. Figure 3(e) shows the response versus voltage and corresponding parabolic fit for the first period of oscillation after the voltage has been applied. Clearly the response deviates significantly from a perfect parabola, however, the measured CPD (i.e. bias value at the maxima) is stable but the error is greatly increased. The error in the fit decays from 17 mV to 1.6 mV in the first millisecond after the pulse is applied. In Fig. 3(f) we plot the residuals of the parabolic fits to the data in Fig. 3(c). The residuals fall off in a similar fashion as is expected for the cantilever ring-down. In summary, the cantilever transfer function gain needs to be accounted for to ensure accurate CPD measurements, and the cantilever ring down (proportional to the cantilever bandwidth) does not affect the measured CPD in G-Mode KPFM and by extension the time resolution, however cantilever bandwidth does strongly influence the error of the fit.

Figure 4 shows experimental data for a tip scanned 50 nm above a gold electrode while 3 *V*_*ac*_ is applied to the cantilever. In addition, a 1 *V*_*dc*_ square wave bias with a period of 2 ms is superimposed on the AC voltage excitation. Figure 4(a–c) depicts the response in the frequency domain, time domain, as well as the reconstructed electrostatic force versus voltage response. In this case the noise floor threshold is used to filter the response before reconstructing back into the time domain but no low, or band pass, filters are used. Clearly the response visualized in both the time domain (see Fig. 4(b)), or against the applied voltage (see Fig. 4(c)), results in deviations from the ideal electrostatic response. In light of the simulations in Fig. 3, we expect contributions from the cantilever dynamics to introduce distortions of the parabola. From the inset of Fig. 4(a) it is clear that both the first and second cantilever eigenmode heavily contribute to the overall time domain response, especially for timescales below the cantilever ringdown. In Fig. 4(d–f) we demonstrate that the influence of the cantilever ring down can be attenuated using appropriate filters. In addition to the noise threshold, we apply a 50 kHz low pass filter in series with a band pass filter (0.1–6 kHz), effectively eliminating the influence of the cantilever eigenmodes and the influence from *1/f* noise. After appropriate filtering, the response tracks the applied potential precisely – and clean undistorted parabolas can be recovered. After correcting for the cantilever transfer function gain the measured CPD was 130 mV +/− 8 mV for the zero bias state and −868 mV +/− 13 mV during the application of +1 V tip bias, see inset of Fig. 4(f), leading to a measured pulse magnitude of 998 mV+/− 15 mV, demonstrating experimentally little or no influence of the cantilever bandwidth on the measured CPD.

In terms of time resolution, for an AC frequency of 15 kHz as used in this work, one period of oscillation is 66 μs, although the *C′*_*z*_ and CPD can be probed at 33 μs intervals (i.e. parabola is recovered for every half period). In standard KPFM, the readout rate is one measurement per pixel – for a pixel time of 4 ms, and an AC frequency of 15 kHz, G-Mode KPFM provides a 120 fold increase in time resolution. We expect that the temporal resolution can be further increased by electrically driving at higher frequencies.

In Fig. 5, we demonstrate the imaging capabilities of G-mode KPFM on a freshly cleaved highly ordered pyrolytic graphite (HOPG) with a partial delamination of the substrate exposing graphene layers, which are electronically decoupled from the graphite surface. Previously, tunneling microscopy and spectroscopy experiments have shown that graphene flakes deposited on graphite after cleaving can be sufficiently decoupled from the substrate to exhibit different structural and electronic properties of the underlying substrate which match that expected of pristine graphene^48^. From Fig. 5, the measured CPD between the pristine and the delaminated areas was approximately 49 mV. Furthermore, fitting coefficients provide maps of the capacitance gradient, which on this sample is strongly affected by step edges. G-Mode KPFM enables reconstruction of the parabolic bias dependence for each pixel, as well as a temporal component of the electrostatic and electrochemical interactions. After fitting, we reduce the 4 GB dataset to maps of *V*_*cpd*_ (*x*, *y*, *t*) and *C´*_*z*_(*x*, *y*, *t*). Whereas this type of bias spectroscopy measurement has been demonstrated in single point mode^3^, or in conjunction with force mapping approaches^3^,^24^, to our knowledge it has never been performed at standard imaging speeds and without DC bias applied to the tip. Multidimensional datasets like these contain rich information about electroactive surfaces or environments such as those recently demonstrated for liquid KPFM, (e.g. electrochemical force microscopy^32^). However, the nature of the data poses a significant problem in terms of visualization^17^. In the next section, we demonstrate how model-free, multivariate statistical analysis can aid in dimensionality reduction and facilitate fast visualization of relevant information.

As described in the previous section, physics-based analysis allows mapping data onto a postulated physical model. At the same time, it is useful to quickly evaluate sample behavior encoded in the raw deflection signal without computationally expensive fitting procedures. Here, we adopt principal component analysis (PCA)^49^, a multivariate statistical approach which separates data into orthonormal components arranged in descending order of statistical significance based on variance content within the dataset. Each principle component consists of an eigenvalue loading map as well as an eigenvector. The data from Fig. 5 was analyzed using PCA on the response signal vs. time for all pixel locations. A more detailed description of the PCA analysis is provided in Supplementary information. The left row in Fig. 6 shows the loading maps corresponding to the first five principal components, and the right row shows their corresponding eigenvectors in the time domain plotted as a function of applied voltage. The color represents time during a single pixel (0–4 ms). It is clearly seen that of the components shown, PCA separates the overall spatial variation into three components. Additionally, the 2^nd^ and 4^th^ eigenvectors, Fig. 6(d,h), show a smooth temporal variation, but the corresponding loading maps have no recognizable spatial correlation (Fig. 6c,g). The smooth time component of the eigenvectors and the lack of spatial order in the loading maps suggest that these principle components are dominated by random experimental error and not indicative of material properties, possibly relating to the fact that the tip-sample distance is not precisely constant but instead electrostatically actuated about the tip sample distance or related to noise in the vertical position feedback. Overall these first five components contain over 98.6% of the statistically relevant information with subsequent principle components being dominated by noise. The loading map corresponding to the first principle component is very similar to the average response, whereas the third and fifth components are characteristic of variation in charge density distribution, (PC3-shift in voltage of the parabola maximum) and variation in the capacitance channel (PC5-shift in amplitude of parabola) shown in the previous section. Here PCA de-correlates the raw signal into electrostatic interactions using purely statistical means with the results matching the physics based approach in this case. For other systems, PCA can highlight and separate more exotic behavior and serves as a good first assessment of the data quality and the range of captured behaviors.

In Fig. 7 we show the results of G-Mode-KPFM across a diode interface. The sample was prepared by cross sectioning a commercial Schottky barrier diode^50^,^51^,^52^. The metal and silicon are clearly identified by differences in topography (Fig. 7(a)). Figure 7(b,c) show the time averaged CPD determined using conventional KPFM and parabolic fitting of G-Mode-KPFM data respectively. Both measurements show similar CPD values, but with a small offset, (43 mV) possibly related to previously reported feedback artifacts^23^. Figure 7(d–f) show the eigenvalue loading maps and the corresponding eigenvectors Fig. 7(g–i). In this case it is clear that PCA separates the response into three distinct regions. Furthermore, three distinct capacitive interactions as shown by the parabolic bias dependence of the eigenvectors. In Fig. 7(f), we clearly observe a region at the interface between the silicon and the metal electrode, approximately 4–5 μm in width showing a more complicated time evolution of the response, demonstrated in Fig. 7(i). We believe this area corresponds to the depletion region, and the measurement frequency corresponds to the frequency interval which is expected to produce large AC transport as has previously been reported for this sample^50^.

G-Mode KPFM is an open loop approach which does not require heterodyne detection or the application of a DC bias. G-Mode KPFM has the ability to probe important information beyond a temporally averaged CPD by capturing and storing a full record of the cantilever dynamics. We demonstrated a range of methods for analysing the resulting multidimensional datasets using physics- and information theory-based approaches. In physics-based analysis, we achieved the direct recovery of the parabolic bias dependence as a function of the applied AC excitation. Fitting of the parabolic bias dependence allowed the CPD and capacitance gradient to be extracted for each half cycle of the modulation voltage. This results in a multidimensional data set containing spatial and temporal dependence of both channels, where the temporal resolution is tied to the frequency of excitation. However even at low frequencies (<15 kHz), temporal resolution is increased by two orders of magnitude over standard KPFM. Therefore, G-Mode KPFM provides the information content of slow spectroscopic methods, but is performed at imaging speeds and retains the temporal response of the measurement without the need for DC bias, making it a promising approach for voltage sensitive materials and operation in the presence of mobile ions (i.e. electrolytes).

This work provides a framework to elucidate details of electrodynamic processes both at solid-gas interfaces on electroactive surfaces and for investigating electrochemical processes at solid-liquid interfaces. We have demonstrated the value of multivariate analysis for fast visualization of the important components of the raw signal using a purely statistical approach and without prior knowledge. Finally, G-Mode KPFM is universally implementable on all AFM platforms and can potentially provide new knowledge on local electrochemical landscapes.

## Methods

### Samples

**Figure 5:**
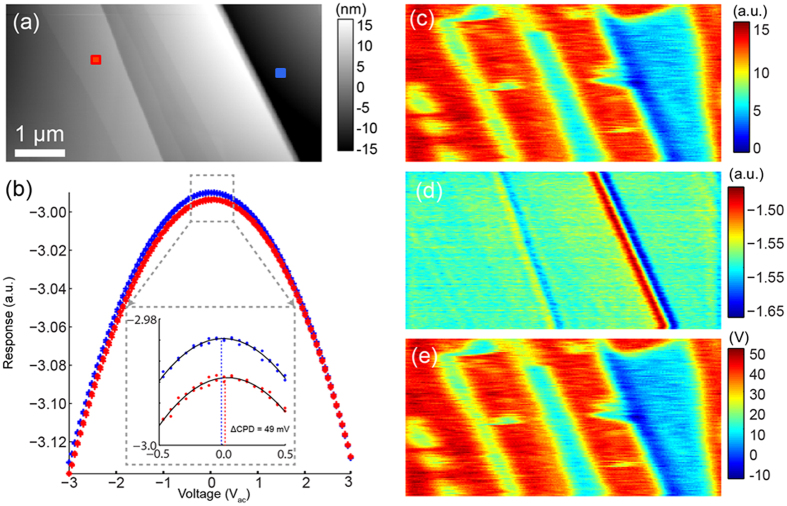
(**a**) Topography image of a HOPG sample. (**b**) Single point parabolas (averaged over the 4 ms pixel time) for two different locations (indicated on (**a**)) showing a 49 mV offset in the CPD between positions. (**c**) Second and (**d**) first order fitting coefficient determined from fitting the parabola at each spatial location for the first period of oscillation. (**e**) The CPD determined from the fitting parameters for the first period of oscillation.

**Figure 6:**
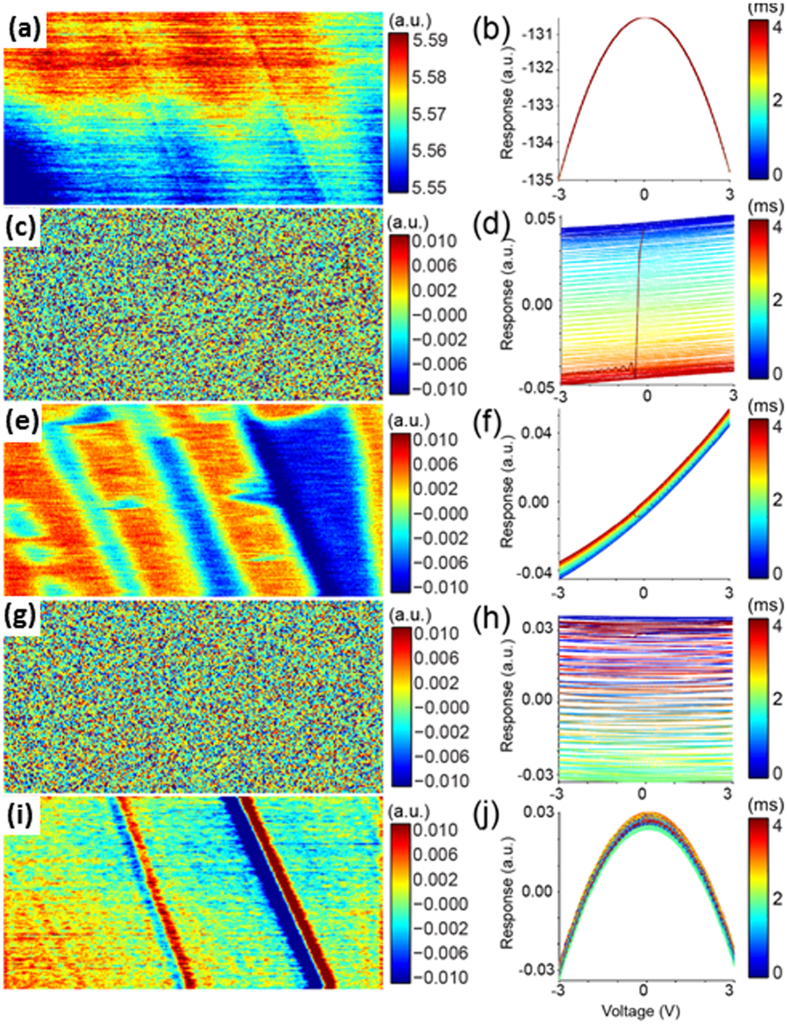
Visualization of the distinct electrostatic interactions using an information based analysis. Left row (**a,c,e,g,i**) depicts the first five principle components in order of rank whereas the right row shows (**b,d,f,h,j**) show the corresponding eigenvalues where the response is plotted as a function of applied voltage. The colormap shows the variation as a function of time (time per pixel = 4 ms).

**Figure 7:**
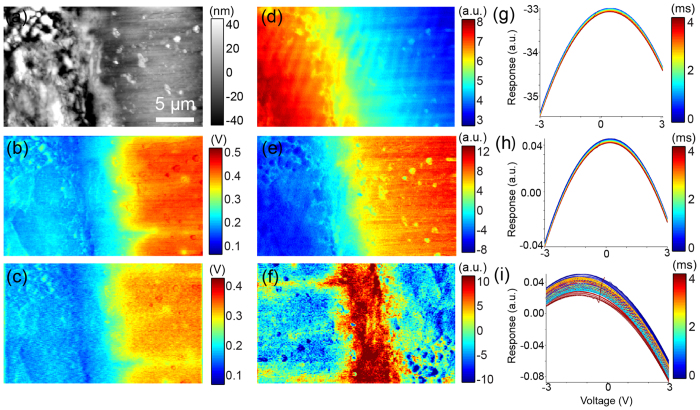
(**a**) Topographical image of a cross section of the Schottky barrier diode. Corresponding time averaged CPD determined using (**b**) classical KPFM (**c**) and G-Mode KPFM. The corresponding PCA loading maps (**d–f**) and eigenvectors (**g–i**) for the first (**d,g**), second (**e,f**) and fifth (**f,i**) principle components.

All samples were mounted on 15 mm steel pucks (Ted Pella) using conductive silver paint (Agar Scientific Ltd.) used to ground the sample with respect to the tip. The charge contrast in the HOPG sample seen in Figs 5 and 6 was only formed after contact mode scanning a rough (e.g. having an unusually large number of step edges) area of a freshly cleaved sample while applying an AC voltage. Prior to scanning the sample in contact no contrast was shown in classical KPFM measurements on the same region (not shown). After contact mode scanning, CPD contrast was observed in both classical and G-Mode KPFM and remained for over a day. We believe the contact mode scanning, delaminated, or partially delaminated, flakes of graphite which were weakly bound after the initial cleaving process. In Fig. 7., the diode sample^50^ was prepared by cross sectioning a commercial Schottky barrier diode. The top of the diode was removed by polishing with diamond paste down to 1 μm gritsize. Further polishing was precluded by selective polishing of the interconnect material, resulting in large topographical variations from the metal to silicon.

### G-Mode KPFM Imaging

Measurements were performed using an Asylum Research, Cypher AFM system with as-received Pt/Ir-coated (Nanosensors, PPP-EFM) cantilevers with a nominal mechanical resonant frequency and spring constant of 75 kHz and 2.8 N/m, respectively. The G-Mode KPFM measurements were performed with a LabView/Matlab controller implemented in PXI architecture using National Instruments NI-6124 fast AWG and DAQ cards.

### Supporting Information.

The Supporting Information is available free of charge on the ACS Publications website at doi: http://pubs.acs.org. Outline of procedure for Automatic Noise Floor Determination. Simulations of G-Mode KPFM performed in Matlab to investigate and demonstrate the correction of, both the cantilever transfer function and the drive/response phase offset on the measured CPD. Information on the application of Principle component analysis to G-Mode KPFM.

## Additional Information

**How to cite this article**: Collins, L. *et al*. Full data acquisition in Kelvin Probe Force Microscopy: Mapping dynamic electric phenomena in real space. *Sci. Rep.* **6**, 30557; doi: 10.1038/srep30557 (2016).

## Supplementary Material

Supplementary Information

## Figures and Tables

**Figure 1:**
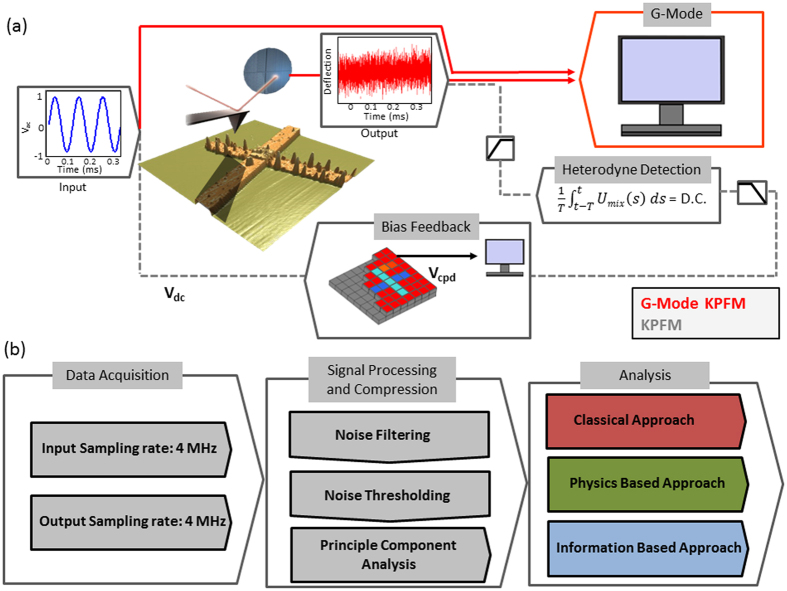
(**a**) Information transfer in conventional KPFM vs G-Mode KPFM. Conventional KPFM (dashed grey line) adopts a combination of heterodyne detection combined with bias feedback to obtain a 2D map of time averaged CPD, whereas G-Mode KPFM streams the entire data deflection signal for offline analysis. (**b**) G-Mode KPFM consists of high speed data acquisition, combined with signal processing and compression as well as a multitude of analytical approaches which can be performed in parallel.

**Figure 2:**
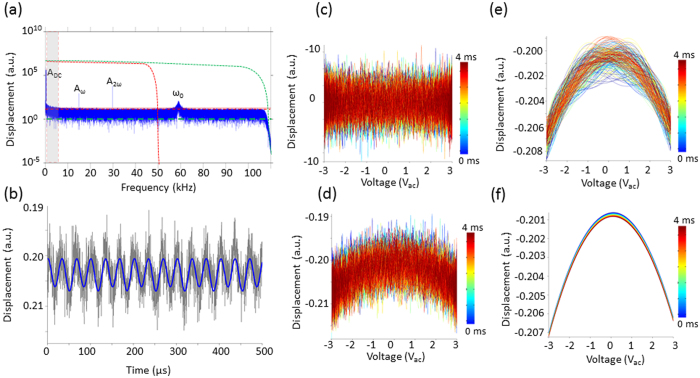
(**a**) Single line of data (∼16,000 points) represented in the frequency domain after the application of a low pass filter of 120 kHz to improve visualization (raw data is 0–2 MHz).(**b**) Raw (grey) and filtered (blue) data shown in the time domain (filters: LPF = 50 kHz (dashed red line in (**a**)); Noise Floor threshold = 10 a.u. (straight red line in (**a**)); Band pass filter = 0.1–6 kHz (grey box in (**a**)). Resulting parabolas obtained from (**c**) raw data (**d**) with 120 kHz LPF and a 1 a.u. noise floor (**e**) 120 kHz LPF and a 10 a.u. noise floor (**f**) 50 kHz LPF, 10 a.u. noise floor and 0.1–6 kHz bandpass filter.

**Figure 3:**
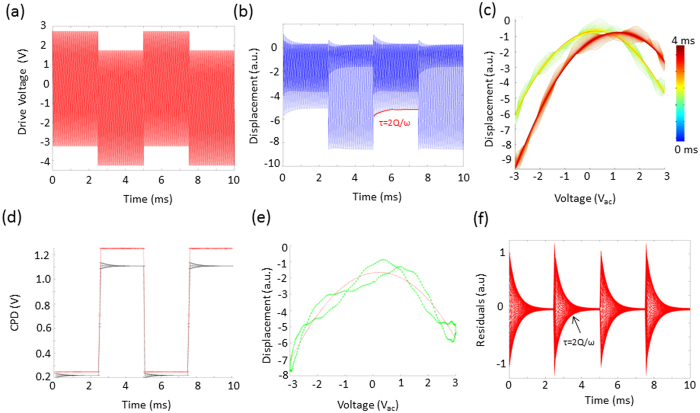
Simulation of the cantilever response and determination of CPD in G-Mode KPFM. (**a**) Excitation waveform (V_ac_ = 2, V_dc_ = −1) (**b**) cantilever response in the time domain and (**c**) reconstructed parabola. (**d**) True (red) and measured (black) CPD before any transfer function is applied. (**e**) Example distorted parabola immediately after the application of the square wave along with a second order polynomial fit (red). (**f**) Residuals of the polynomial fits as a function of time.

**Figure 4:**
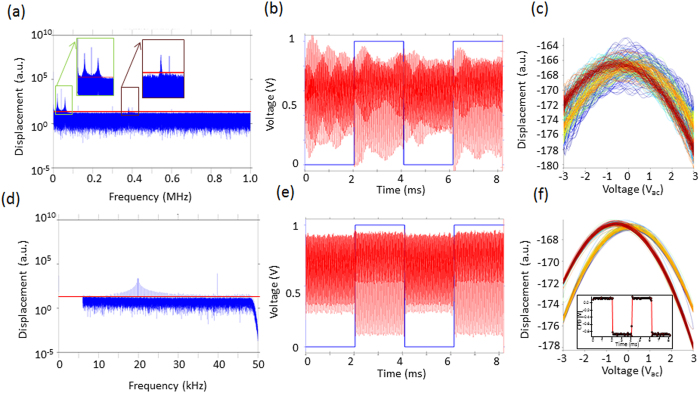
(**a,d**) Single lines worth of data after FFT into the frequency domain. Insets in (**a**) show zoom-in of the first two eigenmode responses. (**d**) Shows the same data after the application of a low pass filter (50 kHz) and a band pass filter (0.1–6 kHz). Corresponding time domain response after the application of the (**b**) noise floor threshold alone (red line) as well as (**e**) after application of low pass and band pass filters. The DC bias applied to the tip is shown in blue. The corresponding parabolas recovered without (**c**) and with (**f**) the use of a low pass filter to remove the influence of the eigenmodes. Inset of (**f**) shows the measured CPD determined from parabolic fitting showing to a relative CPD variation of −998 mV +/−15 mV.
