# Supplementary material for: Full data acquisition in Kelvin Probe Force Microscopy: Mapping dynamic electric phenomena in real space
Source: Sci Rep. 2016 Aug 12;6:30557. doi: 10.1038/srep30557 (PMC4981877; doi:10.1038/srep30557)
Supplement: Supplementary Information [file srep30557-s1.pdf]

Supplementary Information

Full data acquisition in Kelvin Probe Force Microscopy: Mapping dynamic electric phenomena in real space

Liam Collins, [†,‡] *Alex Belianinov,* [†,‡] *Suhas Somnath,* [†,‡] *Nina Balke,* [†,‡] *Sergei V. Kalinin* [†,‡] *and Stephen Jesse,* [†,‡]

[†] Center for Nanophase Materials Sciences, Oak Ridge National Laboratory, Oak Ridge, Tennessee 37831, USA

[‡] Institute for Functional Imaging of Materials, Oak Ridge National Laboratory, Oak Ridge, Tennessee 37831, USA

Automatic Noise Floor Determination

The calculation of the noise floor starts with the assumption that the histogram of the magnitude of the signal exhibits the Rayliegh distribution. Our algorithm iteratively calculates the variance and the threshold of the magnitudes, removes the magnitudes in the signal that are larger than the threshold to arrive at the noise floor. This method uses the following equations:

$$b_j = \sqrt{\frac{\sum_{i=1}^{n} |x_i|^2}{2n}}$$

$$threshold = \sqrt{2b^2 * -\log(tolerance)}$$

Where x_i are the magnitudes of the signal, having n points, in the frequency domain, *tolerance* is the noise floor tolerance (between 0 and 1), j is the iteration number. The algorithm can be summarized as:

1. Calculate *threshold*
2. Set x_i > *threshold* to 0
3. Calculate b_j
4. Calculate $\Delta b = b_j - b_{j-1}$
5. If Δb > 1E-2, repeat steps 1-5, else *threshold* is the noise floor

Simulation of cantilever transfer function

To easily explore the influence of the driving frequency, cantilever transfer function and phase offset on the G-Mode KPFM measurement we developed a model using custom scripts written in Matlab. This allowed us to probe distortions of the real electrostatic force-bias curve due to experimental parameters. In our model the behavior of the cantilever is approximated by two

coupled simple harmonic oscillator (SHO) as given by equation 1 and shown in Figure S1(a) (blue line):

$$A(\omega) = \frac{A1_{\max}\,\omega_1^2}{\sqrt{(\omega^2-\omega_1^2)^2+(\omega\omega_1/Q1)^2}} + \frac{A2_{\max}\,\omega_2^2}{\sqrt{(\omega^2-\omega_2^2)^2+(\omega\omega_2/Q2)^2}} \tag{1}$$

The cantilever parameters for the first and second eigenmode of the cantilever were; $\omega_1 = 75$ kHz, $A1_{max}$=0.95 and Q2 = 50 and $\omega_2 =$ 220 kHz, $A2_{max}$=0.05, and Q2= 100 respectively. To simulate a G-Mode KPFM experiment a single frequency sine wave voltage was used. The resulting electrostatic force was calculated using the formula $F_{\mathrm{el}} = \frac{1}{2}C'z((V_{dc} - V_{CPD}) + V_{ac}\sin(\omega t))^2$ where V_{ac} = 2, V_{dc}=0; V_{cpd}=0.3V and $C'z = 1.$ Figure S1(a) shows the resulting electrostatic force when the drive frequency is 15 kHz having a DC, first and second harmonic response. The simulated electrostatic force is plotted with added noise using a red line in Figure S1(a). The cantilever response was then found by the product of the cantilever transfer function and the electrostatic force in the frequency domain. Figure S2(b) shows the applied voltage and resultant cantilever response in the time domain. When the cantilever response is plotted against the applied voltage, as shown in Figure S1(c), the full response vs voltage can be recovered for each half cycle of the applied voltage. Fitting (red line) was performed using a second order polynomial curve described by $\mathrm{y} = \mathrm{a}x^3 + bx + c$, where CPD = -b/a, a is directly proportional to C' and c is the offset of the parabola.

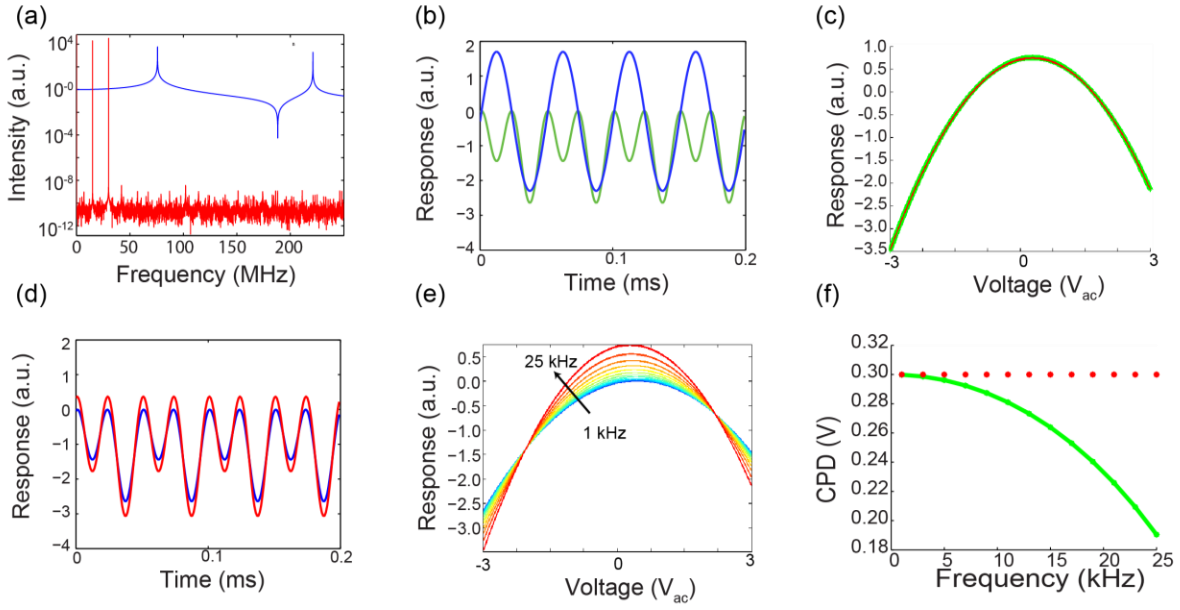

Supplementary Figure 1 | Influence of Cantilever transfer function in G-Mode KPFM. (a)The modelled SHO transfer function of the cantilever (blue) and the applied electrostatic force (red). (b) The resulting electrostatic excitation (blue) and electrostatic response (green) wave in the time domain. (c) Recovery and fitting of the parabolic electrostatic force from the data in figure (b). (d) The electrostatic response with (red) and without (without) the influence of the cantilever transfer function. (e) Distortions of the parabola (blue-1 KHz: red-25 KHz) and (f) the CPD as a function of frequency when the cantilever transfer function is considered.

Figure S1(d) depicts the influence of the cantilever transfer function on the measured response. The difference between the electrostatic force (red) and the measured response (blue) are due to different harmonics having different amplification due to their proximity to the resonance frequency. Figure S2 (e) shows how the influence of the drive frequency on the parabola due the cantilever transfer function distorts the shape of the restored parabola. The parabola becomes increasingly distorted with increasing frequency due to the un-proportional amplification of the dynamic response at the harmonics near the resonance frequency. Figure S2(f) shows how the CPD, determined from fitting the data in Figure S2(e). This demonstrates that if the cantilever transfer function is not correctly taken into account the resulting CPD values can significantly deviate from the actual physical values. Note that this is inherent in all open loop dual harmonic approaches. In the case of G-Mode KPFM, since the output contains a permanent recorded of the entire cantilever spectrum, a calibration of the transfer can be performed by fitting the

resonance peak to a SHO model described above and determining the frequency dependencies of transfer function parameters. This method has previously been described elsewhere. [1]

Simulation of Phase offset

Another equally important consideration is the phase offset between the excitation waveform and the response. In standard KPFM, it is critical to correctly adjust the phase offset of the Lock in amplifier so that the response is either precisely in phase (or out of phase) with the driving voltage. This is performed by the user at the beginning of each measurement, as a calibration stage and is assumed to remain constant throughout the measurement. Unfortunately this is unverifiable when the bias feedback loop is closed and it is recommended to ensure correct phase offset several times during the data collection process.[2]

Similar, in G-Mode KPFM it is equally important to correctly adjust the phase offset between drive and response. Figure S2 depicts the effect of phase offsets in the measurement as well as the ability to correct the hysteresis resulting from any phases offset. Unlike standard KPFM, in G-mode KPFM which is an open loop technique we can determine what the phase offset at each point in time (or more precisely for each period of oscillation) of our measurement by performing a software based Lock in amplifier.[3,4] Once the phase offset is determined, we can phase shift the response by the appropriate offset and recover the undistorted parabolic bias dependence of the electrostatic force. Correctly accounting for both phase and the transfer function of the cantilever allows for accurate and quantitative extraction of electrostatic and electrochemical parameters.

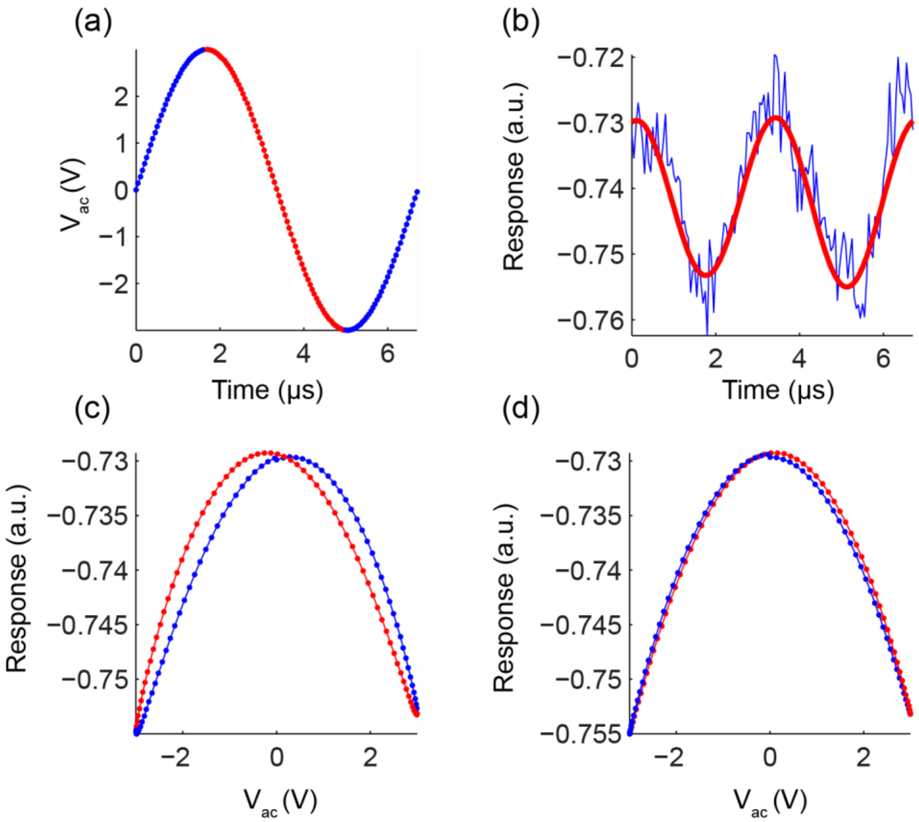

Supplementary Figure 2 | Influence of Phase offset in G-Mode KPFM. (a)The applied voltage is shown where the red and blue colors indicate positive (blue) and negative (red) polarity of the waveform respectively. (b) Shows the raw (blue) and processed (red) cantilever response to the applied voltage in (a). (c) Shows the electrostatic force-voltage curve recovered by plotting the response versus the applied bias. (d) Clear hysteresis between the positive and negative polarity can be observed. After shifting the response by the phase offset determined using a LIA algorithum the hysteresis was almost completely eliminated.

Principle Component Analysis

Principal component analysis performs a principal axis rotation of the variance–covariance matrix of the original dataset and converted into a linear superposition of orthogonal, linearly uncorrelated eigenvectors. The direction cosines between the new and old axes are eigenvectors calculated by the singular value decomposition of the variance–covariance matrix, C =

AA^T where A is the matrix of all experimental data points A_{ij}, i.e., the rows of A correspond to individual grid points i = 1, . . . and columns correspond to voltage points, j = 1,. . . ..

The resulting variables, or principal components, are orthogonal and as such uncorrelated, and are arranged such that corresponding eigenvalues are placed in descending order, by variance. The values for these new variables are the principal component eigenvalues, and the relationship between the original bias dependent response and the new bias dependence variables is the principal component loading. The variance captured in each principal component is expressed as the fraction of the eigenvalue divided by the sum of the eigenvalues for all the principal components in the model. This is convenient when the components of interest are identified and the dataset reconstructed using this fractional information – acting as an information based filter. Principal component loading images were constructed by plotting the score value of each pixel in X–Y space.

1 Collins, L. *et al.* Open loop Kelvin probe force microscopy with single and multi-frequencexcitation. *Nanotechnology* **24**, 475702, (2013).

2 Jacobs, H., Knapp, H. & Stemmer, A. Practical aspects of Kelvin probe force microscopy. *Review of Scientific Instruments* **70**, 1756-1760, (1999).

3 Collins, L. *et al.* Multifrequency spectrum analysis using fully digital G Mode-Kelvin probe force microscopy. *Nanotechnology* **27**, 105706, (2016).

4 Collins, L. *et al.* G-mode magnetic force microscopy: Separating magnetic and electrostatic interactions using big data analytics. *Appl. Phys. Lett.* **108**, 193103, (2016).
